# The Influence of the Steam Sterilization Process on Selected Properties of Polymer Samples Produced in MEX and JMT Processes

**DOI:** 10.3390/ma17235763

**Published:** 2024-11-25

**Authors:** Małgorzata Zaborniak, Janusz Kluczyński, Jakub Stańko, Tomasz Ślęzak

**Affiliations:** 1 Faculty of Mechanical Engineering and Aeronautics, Rzeszow University of Technology, Powstańcow Warszawy 8 St., 35-959 Rzeszow, Poland; mzab@prz.edu.pl (M.Z.); 164259@stud.prz.edu.pl (J.S.); 2 Institute of Robots and Machines Design, Faculty of Mechanical Engineering, Military University of Technology, gen. Sylwestra Kaliskiego 2 St., 00-908 Warsaw, Poland; tomasz.slezak@wat.edu.pl

**Keywords:** polymer material study, additive manufacturing technologies, 3D printing, sterilization, strength testing

## Abstract

Polymeric materials are widely used in medical engineering, and with the dynamic development of additive manufacturing (AM) technology, increasing attention is being paid to research on the mechanical strength of composite polymer structures. At the same time, the impact of sterilization on, for example, surgical templates and the influence of the sterilization process on the geometry of these parts have not been sufficiently studied. In this work, the effect of steam sterilization on samples made of polymer materials for medical applications was presented. This research was carried out on samples with normative geometry made of polyetheretherketone (PEEK) polymers produced using the Material Extrusion (MEX) AM process and acrylic formulation (MED610) produced by Jetting Modeling Technology (JMT). These materials provide biocompatibility, which makes them suitable for potential medical applications. Steam sterilization was performed in an autoclave at temperatures of 121 °C and 134 °C. The three-point bending strength properties were determined according to ISO 178 standards. An INSTRON 5967 strength testing machine was used for those tests. Surface roughness analysis (according to ISO 21920) was performed and presented in 2D and 3D surface views using the Mountains Map Software (version 6.0).

## 1. Introduction

The application of additive manufacturing (AM) in medical engineering is constantly evolving. Initially, these were primarily 3D objects for preoperative visualization, surgical templates, implants, or lattice structures for tissue engineering applications (so-called scaffolds). Over the last decade, there has been a significant increase in the number of implantable medical products. Currently, AM, commonly referred to as 3D printing, is also used for drug delivery systems. In response to increased interest from the medical industry and widespread access to 3D printers, there is a growing need for research on the utility of polymeric materials for medical applications. Before 3D printing can be widely used, for example, for the regeneration of complex tissues (e.g., bones, muscles, vessels, and nerves in the craniofacial area) and complex organs with intricate 3D microarchitecture (e.g., liver and lymphatic organs), it is necessary to determine the technological limitations of the AM methods used precisely. There are many scientific studies related to the applications of 3D printed devices in medicine available. However, the exact effects of sterilization techniques on biopolymer properties remain a subject of discussion in the field of biomedical engineering, especially currently with the development of devices printed using 3D printing technology [1,2]. Despite the large number of research findings in polymer printing literature on the technology [3,4,5], relatively few studies focus on the impact of sterilization procedures on their properties, which is one of the crucial preparation processes that allows proper preparation of the part dedicated to medical application.

Sterilization is one of the standard processes applied to surgical templates or implants. This procedure is mandatory when using medical objects manufactured with AM technologies in the operating room, confirming the importance of analyzing the effects of the sterilization process on the geometry of additively manufactured parts and changes in their surface properties after the sterilization process. Conventional sterilization techniques, such as high temperature, gamma radiation, and ethylene oxide, can cause damage, alteration, or release of toxic chemicals due to the thermal and hydrolytic sensitivity of commonly used materials [6]. There is no single standard method with universal parameters for sterilizing implantable medical products based on polymers. The methods and parameters of the AM process must be optimized depending on the type of materials subjected to sterilization. In addition to their effectiveness, sterilization methods should not cause significant changes in the physical, chemical, mechanical, and biocompatibility properties of the material. Despite significant progress in the development of biomaterials in recent years, sterilization techniques have remained unchanged over the years. Currently, there are three conventional sterilization methods for medical products commonly used in the industry: chemical sterilization using ethylene oxide (EO), gamma irradiation, and steam sterilization. Unfortunately, for sterilizing polymer materials with limited thermal and chemical resistance, these conventional techniques have certain drawbacks that can seriously alter their original properties. It has been widely demonstrated that steam sterilization is not suitable for thermally and hydrolytically sensitive biomaterials. Other sterilization techniques, such as hydrogen peroxide gas plasma, peracetic acid, or ozone treatment, are also being investigated as alternatives to conventional techniques [7,8,9].

The availability of studies that analyze the impact of the sterilization process on polymer materials is limited. For example, the impact of sterilization on 3D-printed paper models has been studied in [2]. The effect of sterilization on objects printed using PolyJet technology (Stratasys, Eden Prairie, MN, USA) was investigated. In all cases, the researchers concluded that disinfection of AMed objects should be limited [1]. Therefore, research in recent years has focused on innovative sterilization processes for polymer materials and on minimizing the impact of the sterilization process on material integrity. In available studies [1,2], it has been shown, among other things, that sterilization of dental objects intended for clinical use can lead to deformation of the printed model, especially in the case of thermal sterilization.

However, these conclusions contradict the studies available from other authors who found that objects on MEX 3D printers can be sterilized in an autoclave [10]. The main limitation of these printing methods is the sensitivity of the AMed bio models to temperature, which almost precludes their sterilization using conventional methods. One of the most promising materials that is becoming more and more popular in medical applications is polyetheretherketone (PEEK) due to its good mechanical properties, high wear resistance, and elastic modulus that is almost equal in value to human bones [11]. The most important factor that makes this material valuable for medical applications is its homogeneity and chemical compatibility with a lot of bioactive substances. What is more, PEEK is characterized by strong responsiveness to sterilization [12]. A characteristic structure (surface roughness) of the AMed material after the MEX process increases its bioactivity [13]. At the same time, the advantages of AM technologies (the possibility of getting geometrically complex parts) could cause a deformation of the parts of the volume that have a small value of wall-thickness [14]. As was analyzed by Limaye et al. [15], it is very important to ensure the porous structure of the PEEK-based parts for medical applications. Such kind of structures could have a small wall thickness, which could make them more resistant to some kinds of sterilizations where penetration of the sterilizing medium could be limited. On the other hand, there is available more precise AM technology for resins—JMT. One of the most popular materials dedicated to medical applications is an andacrylic formulation (MED610). Due to its limited biocompatibility (short-term mucosal-membrane contact of up to 24 h [16]), it is mostly used for surgery tool production that is characterized by complex geometry [17]. The JMT technology is dedicated to AM production with significant precision and low surface roughness which have a great influence on the wear properties of the parts produced by JMT [18]. Those kinds of factors (dimensional accuracy, surface roughness, etc.) could be dependent on the sterilization method that has not been properly analyzed in the available literature.

The biocompatible material PEEK belongs to the family of polyaryletherketone (PAEK) polymers and has a wide range of applications. The material is thermoplastic and has high mechanical strength and excellent thermal properties. It is characterized by high chemical stability, corrosion resistance, and excellent resistance to extreme temperatures. Compared to titanium commonly used in implant production, it exhibits better load distribution and lower stiffness, making it a good material alternative, for example, during spinal implantation. Due to its properties, achieving precise dimensions and high print quality requires appropriately selected manufacturing process parameters for PEEK, including high extrusion temperatures, heated beds, and heat-retaining printing chambers. The AM technique used in its extrusion utilizes support structures which, due to PEEK’s properties, are challenging to remove. The MED610 material is a polymer developed specifically for medical applications, such as the production of prototypes, anatomical elements, training aids, and tools. It is designed to comply with biocompatibility and medical safety standards, making it suitable for use in human body contacts. It is optically transparent, enabling visualization of the interior, which allows precise structures and usefulness, for example, in surgical planning. 

Continuous development of polymer materials dedicated to AM leads to the development of biocompatible materials. Therefore, this work aims to analyze the impact of temperature sterilization on selected functional properties of AM parts made of high-temperature-resistant PEEK and MED610 materials.

## 2. Materials and Methods

### 2.1. Materials

In the field of AM implants, a variety of materials are used, each distinguished by its functional properties, including biocompatibility and broadly understood strength properties. This research was carried out on samples with standard geometry, manufactured using the MEX additive process with PEEK-based filament (3DGence Ltd., Przyszowice, Poland) and the Jetting Modeling Technology (JMT) with MED610—Stratasys (Stratasys Inc., Eden Prairie, MN, USA). Table 1 presents selected mechanical properties of the polymer materials used.

### 2.2. AM Process

To obtain PEEK samples, an industrial fused filament fabrication (FFF) system was used by 3DGence Industry F421 (3DGence Ltd., Przyszowice, Poland). In the case of MED610, samples were printed on a Stratasys Object CONNEX3 (Stratasys Inc., Eden Prairie, MN, USA) device. The printed shape for both materials had supports that were removed in a pressure washer after the printing process. Process parameters used for each sample group are shown in Table 2.

### 2.3. Steam Sterilization

**Figure 1:**
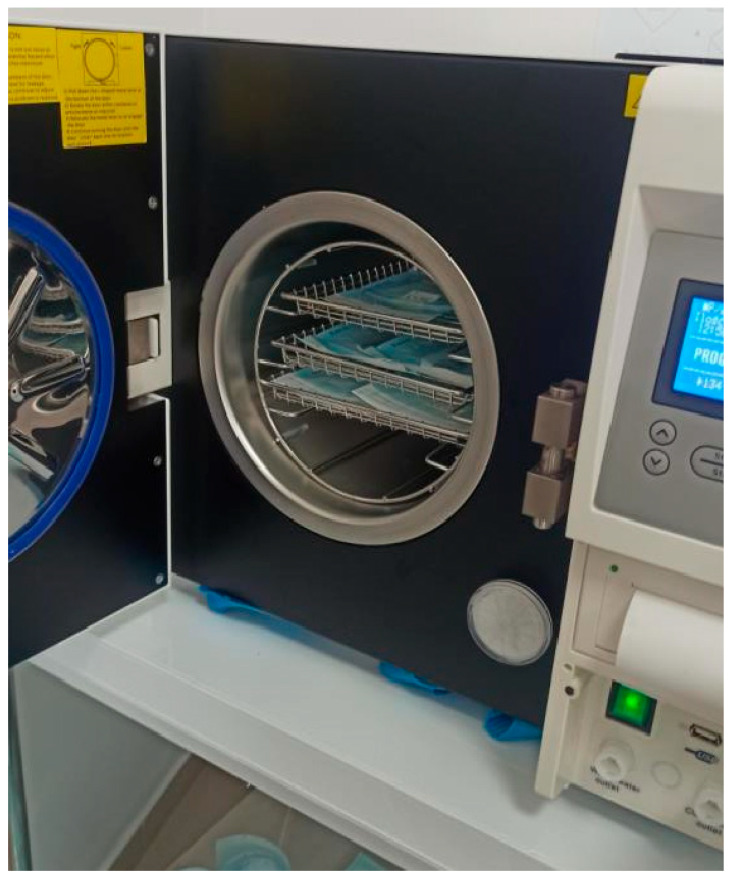
The autoclave chamber with the prepared samples.

The main aim of this research is to investigate the effect of the steam sterilization process, carried out in an autoclave at temperatures of 121 °C and 134 °C, on selected properties of the developed samples. The experimental studies required the development of the sterilization process and were conducted using an autoclave from the E 12L BLACK IS YE-SON series (IMPALL Rozwandowicz Bocheski Sp.K., Łódź, Poland), shown in (Figure 1). Uniform process conditions were applied to all samples, enabling a comparative analysis of the results obtained. Sterile high-temperature sterilization is the process of eliminating microorganisms at high temperatures. The most commonly used method is autoclaving (steam sterilization under pressure). The method involves using high steam pressure at a temperature of approximately 121 °C or 134 °C, which effectively kills bacteria, viruses, and fungi. The sterilization process using saturated steam for porous products according to ISO 17665-1:2006 [21] is divided into 6 main phases. Currently, two basic programs are standard in modern sterilizers:

Temperature: 121 °C → sterilization time: about 15 min.
Temperature: 134 °C → sterilization time: about 3 min.

The lower the temperature, the longer the process takes.

After the sterilization, the samples were subjected to standard medical procedures—cooling and storage at room temperature.

### 2.4. Surface Roughness Analysis

Surface roughness is an important parameter when it comes to evaluating the quality of material surface manufacturing. Measurement of surface roughness can help to determine whether surfaces meet industrial standards and safety requirements regarding performance, as well as help to track process variables that may affect surface characteristics, such as aging, chemical deposition, or mechanical wear.

Material samples, both in their post-manufacture state and following medical sterilization, were analyzed for surface characteristics and subjected to static three-point bending tests. In AM, essential parameters include post-sterilization dimensional accuracy, material resistance to chemical or thermal changes, and surface properties, such as roughness, topography, and geometry, all of which influence human tissue interactions [22,23,24,25,26]. For instance, surface characteristics of surgical templates and implants directly affect tissue responses. Increased surface texture promotes osseointegration between tissues and implants, making surface testing essential to evaluate biocompatible material properties, particularly for medical applications like implants. Examining surface texture in this field is multi-dimensional. From a biocompatibility perspective, smoother surfaces may reduce tissue irritation or inflammation, positively impacting material compatibility with human tissues. The relationship between material surfaces and biological tissues is directly linked to surface structure, as excess roughness can increase wear and undesirable tissue reactions. Notably, surface properties impact cell adhesion and proliferation, critical for cellular processes, which underscores the importance of determining optimal surface texture for cell attachment in implants and prosthetics [1,27,28,29].

**Figure 2:**
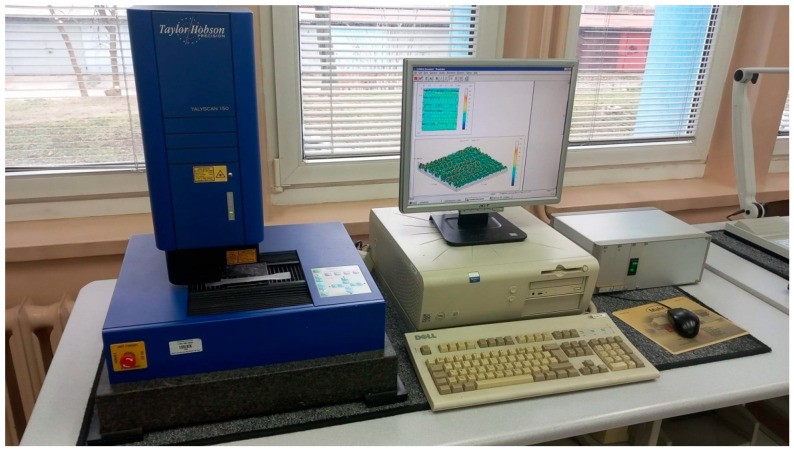
Set up with the Talyscan 150 3D profilometer.

Furthermore, rough surfaces may facilitate bacterial adhesion, increasing the risk of biofilm formation, a significant concern as biofilms on medical devices contribute to infection risks. Thus, controlling surface texture assists in developing materials resistant to bacterial growth. Clinical research has widely shown that implants with osteophilic surfaces integrate more quickly and strongly with bone tissue than those with traditional surfaces. For example, an implant with an osteophilic surface can achieve full integration with hard tissue in a few weeks, whereas implants with conventional surfaces may require up to six months to fully integrate with bone. The 2D surface roughness parameters were measured on the surface using a MarSurf M300 profilometer (Mahr GmbH, Göttingen, Germany). Based on these measurements, the cutoff length value λ_c_ was determined according to the procedure outlined in the ISO 21920-1 standard [30]. The evaluation of 3D parameters was conducted using a Talyscan 150 3D profilometer (Taylor Hobson Ltd., Leicester, UK) (Figure 2).

During measurement, the minimum available measurement speed of 2000 µm/s was applied. During the determination of the surface roughness parameters, a filtering process was conducted, which initially involved the removal of the shape deviations obtained. This was performed using a first-degree polynomial. Subsequently, to separate the long-wave components, a Gaussian filter with λ_c_ = 0.8 mm was applied, which marks the transition from roughness to waviness. As a result, a three-dimensional visualization of surface roughness was obtained. The ISO 21920 standard interprets the surface roughness parameters obtained from measurements as follows:

(a) S_a_, whose value is determined as the arithmetic mean of the absolute values of the ordinate values within the defined area:
  (1) $$S_{a}=\frac{1}{A}\iint_{A}\left|z\left(x,y\right)\right|dxdy$$
(b) S_q_, whose value is determined as the root mean square of the ordinate values within the defined area:
  (2) $$S_{q}=\frac{1}{A}\iint_{A}\left.\left|z^{2}(x,y)\right.\right|dxdy$$
(c) S_v_ is the smallest value of the depth within the defined area, while the parameter S_p_ is the largest value of the height within the defined area. Sz is expressed as the sum of the maximum height of elevation and the maximum depth height within the defined area:
  (3) $$S_{z}=S_{v}+S_{p}$$
(d) S_sk_ is the ratio of the mean value of the third power of the coordinates and the third power of S_q_ within the defined area:
  (4) $$S_{sk}=\frac{1}{S_{q}^{3}}\left[\frac{1}{A}\iint_{A}z^{3}(x,y)dxdy\right]$$
(e) S_ku_ is the ratio of the mean value of the fourth power of the ordinates and the fourth power of S_q_ within the defined area:
  (5) $$S_{ku}=\frac{1}{S_{q}^{4}}\left[\frac{1}{A}\iint_{A}z^{4}(x,y)dxdy\right]$$

### 2.5. Bending Tests

**Figure 3:**
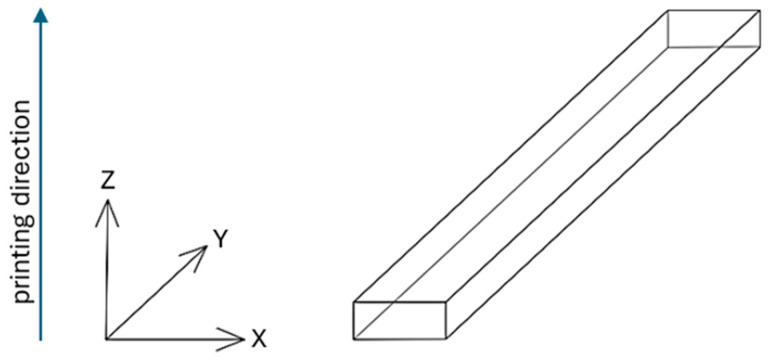
Visualization of a test sample during the 3d printing process along the “Y” axis.

The MultiTest-dV 2.5 device (Mecmesin Ltd., Slinfold, UK) was used to perform measurements in a static three-point bending test. This testing equipment is designed to carry out tensile and compression tests. In accordance with the ISO 179-1:2010 standard [31], the samples were designed using CATIA software (version V6 R22). Each sample was a rectangular prism with dimensions of 4 mm × 10 mm × 100 mm. The 3D model was saved in STL format and then imported into CAM software, which was specifically used for preparing additive manufacturing (AM) processes. The samples were printed in one orientation as illustrated in Figure 3. The test samples were subjected to three-point bending. For each combination, at least 5 samples were tested. The testing for all samples was carried out until their deflection reached 1.5 times their thickness.

## 3. Results and Discussion

### 3.1. Surface Roughness Measurements of the Test Samples

**Figure 4:**
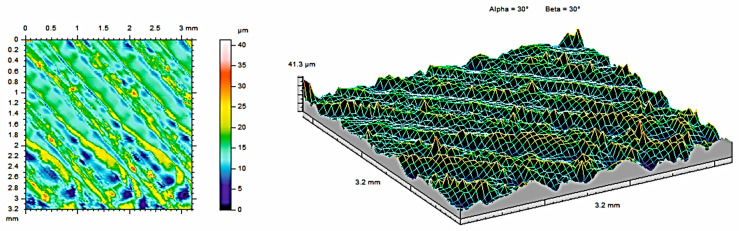
Surface roughness maps obtained from measurements for the sample made of PEEK material without sterilization.

**Figure 5:**
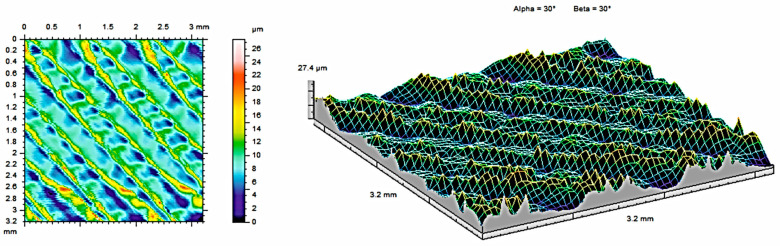
Surface roughness maps obtained from measurements for the sample made of PEEK material after sterilization with steam at 121 °C.

**Figure 6:**
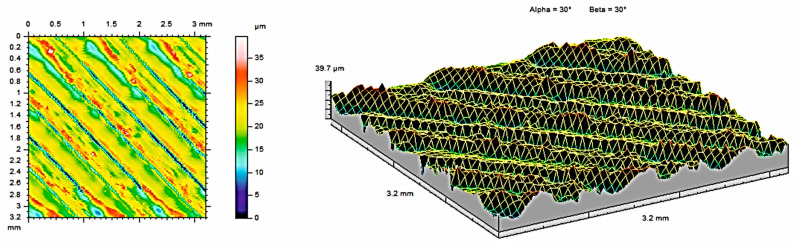
Surface roughness maps obtained from measurements for the sample made of PEEK material after sterilization with steam at 134 °C.

The analysis of contour maps and isometric surface images of the tested samples revealed certain surface characteristics that influence the functional properties of the surface. All the measurements were made on the same areas after each condition (as built, after 121 °C, and after 134 °C. To obtain the highest, possible amount of data about the surface, the highest possible measurement area was analyzed for each sample (3.2mm × 3.2mm). Each area was selected from the middle of each material sample and was subjected to measurements at each condition. Such an approach allows for obtaining the most, possible precise results subjected only to an error of the measurement head (equal to ±0.15 µm). The appearance of the topography was visually analyzed. Figure 4 shows contour maps and isometric images of samples made of PEEK material, while Figure 5 and Figure 6 show contour maps and an isometric image of the same samples subjected to additional steam sterilization. Visual examination of the images revealed differences between the surfaces, confirming the observations of the coverage area and the mentioned parameters. Numerous unevenly distributed material bands can be observed on the surface. This is associated with the characteristic of the 3D printing process, where the directions of the bands seen in Figure 4 are consistent with the direction of track deposition during the MEX process. Additionally, discoloration was observed on the surface subjected to steam sterilization. During the sterilization process at elevated temperatures, reactions occur that affect the increase in band intensity.

Table 3 presents the surface roughness parameters obtained from the measurements for the PEEK sample. Compared to non-sterilized samples, parts that were subjected to sterilization at 121 °C show lower average roughness (S_a_) and shallower indentations (S_v_). The S_sk_ parameter provides information about surface asymmetry and is useful for monitoring various types of wear conditions on the surface of the tested part. The value of the S_sk_ parameter indicates a predominance of peaks over valleys for S_sk_ < 0. When the skewness values of S_sk_ are negative, most of the material is located near the surface peaks. The S_ku_ parameter indicates the presence of unexpectedly high peaks or deep valleys on the surface for S_ku_ > 3. Surface wear reduces the value of S_sk_.

**Figure 7:**
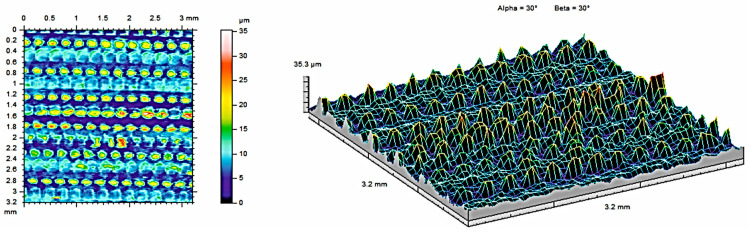
Surface roughness maps obtained from measurements for a sample made of MED610 material without sterilization.

**Figure 8:**
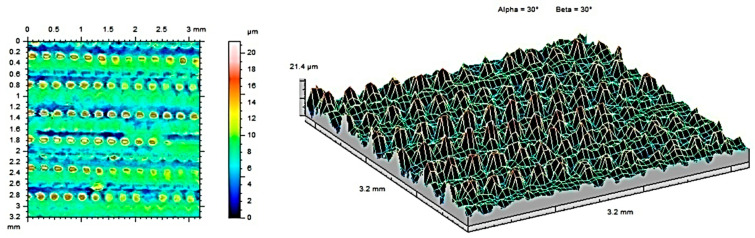
Surface roughness maps obtained from measurements for a sample made of MED610 material after sterilization with steam at 121 °C.

**Figure 9:**
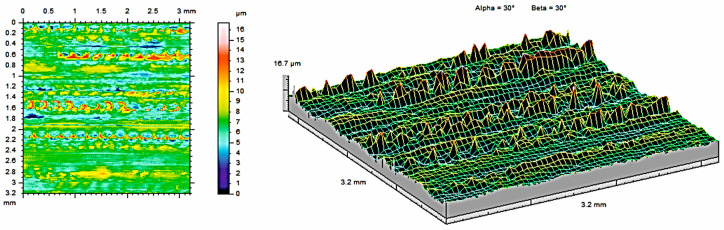
Surface roughness maps obtained from measurements for a sample made of MED610 material after sterilization with steam at 134 °C.

The surfaces of the sample sterilized at 134 °C were observed to have evenly distributed peaks, confirming their anisotropic characteristics. Figure 7 shows contour maps and isometric images of a sample made of MED610 material. Figure 8 and Figure 9 show contour maps and isometric images of the MED610 material sample after steam sterilization. Visual analysis of the images reveals differences between the surfaces. The surfaces analyzed are characterized by uneven material distribution. The geometric structure of the surface is determined by the manufacturing method and the sterilization process performed. The arrangement of characteristic processing traces on the surface, their organization, or randomness is determined by the degree of isotropy of the geometric surface structure, which in the analyzed case is almost uniform in all directions. In the case of methods that utilize volume photopolymerization, a uniform internal structure of the samples can be observed [5], resulting in a reduced influence of the direction of layer deposition during the additive building process of the manufactured part. However, this positive effect decreases with increasing sterilization temperature.

Table 4 presents the surface roughness parameters obtained from the measurements for the MED610 samples. Compared to non-sterilized samples, the sample subjected to sterilization at 134 °C exhibits the lowest average roughness (S_a_) and shallower depressions (S_v_).

**Figure 10:**
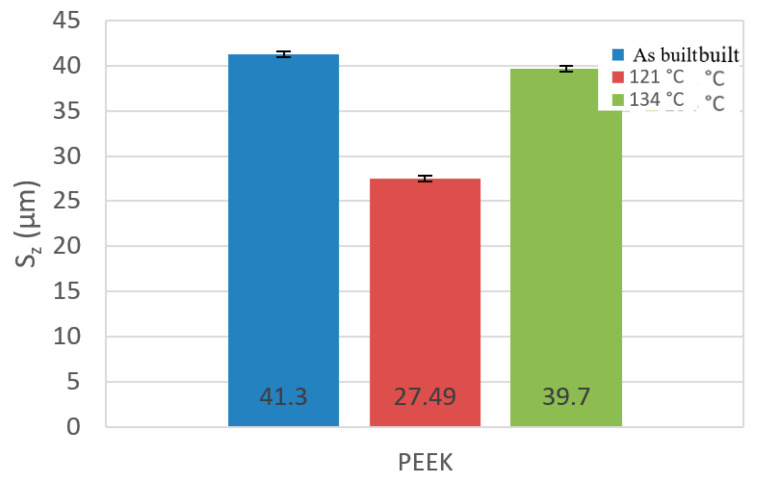
A column chart depicting the values of the parameter S_z_ for samples made of PEEK material.

**Figure 11:**
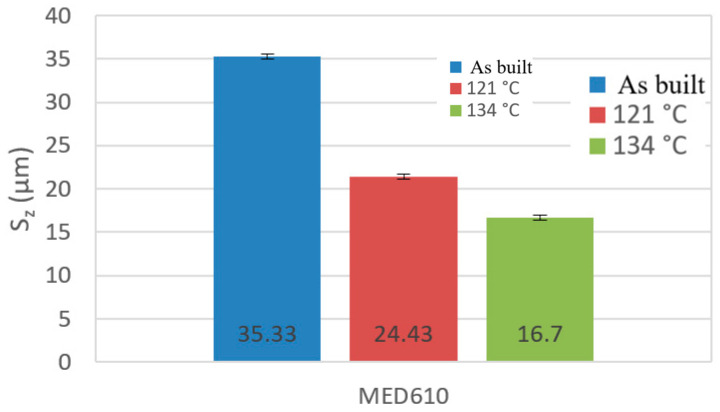
A column chart depicting the values of the S_z_ parameter for the MED610 material.

The two- and three-dimensional visualizations (Figure 3, Figure 4, Figure 5, Figure 6, Figure 7 and Figure 8) depict the surface characteristics measured using the parameter S_z_, which expresses the sum of the maximum peak height and the maximum valley depth. A comparative analysis of the S_z_ parameter was performed for the tested samples, as illustrated in Figure 10 and Figure 11.

In the case of the PEEK material (Figure 10), the sample not subjected to sterilization exhibited the highest value of the S_z_ parameter, while the sample sterilized at 121 °C showed the lowest value. The reason for this result is the duration of sterilization. The sterilization process reduces the S_z_ value in both methods compared to the sample not subjected to sterilization. The sample achieves a smaller value of the S_z_ parameter, measuring 27.49 µm, achieved by the sample with a sterilization duration of 15 min, as opposed to the process at a temperature of 134 °C, which lasted 3 min.

The S_z_ parameter for the MED610 material samples (Figure 11) decreased as a result of the sterilization processes. With increasing temperature, the value of the S_z_ parameter decreased. Upon observing this relationship, we can conclude that the melting temperature of this material is close to the temperature that prevails in the autoclave chamber during the sterilization process, making the surface of the peaks and valleys on the material less visible.

### 3.2. Strength Testing Was Carried out Using a Static Three-Point Bending Test

**Figure 12:**
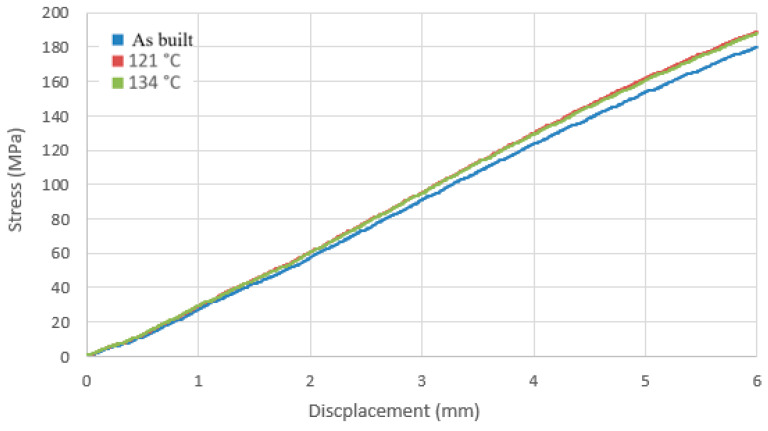
Stress vs. displacement curve for samples made of PEEK polymer.

**Figure 13:**
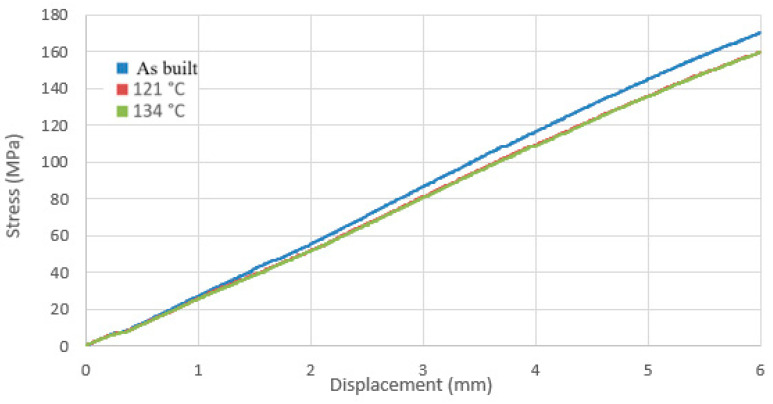
Stress vs. displacement curve for samples made of MED610 polymer.

The results obtained from the static three-point bending test were grouped according to the material used, and graphs (Figure 12 and Figure 13) were created illustrating the relationship between bending stresses and sample deflection values of the sample under the influence of applied force.

In the case of PEEK polymer samples, prints subjected to sterilization processes exhibited higher bending stress values during the bending test compared to those that were not sterilized. This indicates that differences in geometry resulting from temperature variations increase the stresses present in the PEEK material.

The samples made of the MED610 material exhibited opposite results (Figure 13) compared to the material tested material. Sterilization caused a reduction in the stress that occurred compared to the sample not subjected to this process. Taking into account these data, the differences in geometry resulting from temperature reduce the stresses present in the MED610 material [24,32].

## 4. Conclusions

The research conducted has shown that sterilization processes affect surface properties, causing changes in surface roughness. The materials examined exhibit different reactions to the sterilization process. In the case of samples made of PEEK material subjected to sterilization, stress values increase. The opposite characteristic is exhibited in samples made of MED610 material, where the sterilization process reduces the bending stresses. Additionally, MED610 exhibits high mechanical strength and can be used to manufacture even very delicate details. At the same time, it is flexible, facilitating manipulation and use of printed elements. Its continuous contact with the skin is limited to a maximum of 30 days, while in the case of mucous membranes, it is limited to 24 h [16]. This means that it can be temporarily implanted in a patient’s body but cannot remain permanently implanted. In this case, it will be required to withstand the sterilization process, which is demonstrated for various decontamination methods. When considering the use of PEEK and MED610 as temporary implants in the human body, the relationship between surface roughness and bending strength becomes particularly relevant. For PEEK, rougher surfaces after sterilization are combined with increased bending strength, which is beneficial in implant applications. Increased surface roughness promotes better osseointegration, as rough surfaces encourage tissue attachment and stability within the body. For MED610, smoother surfaces resulting from sterilization are combined with slightly lower bending strength, potentially limiting its suitability for load-bearing applications as temporary implants. Smoother surfaces in MED610 could be advantageous in specific contexts, such as reducing friction and minimizing irritation in soft tissue applications. However, lower bending strength may compromise its durability and resilience when subjected to bodily stresses. For MED610 to be used effectively in temporary implants, it would need to be applied in areas with minimal mechanical load or in applications where flexibility is more critical than high structural strength.

These studies constitute a stage of broader strength research carried out by the team, which includes fatigue strength tests and creep tests during simple bending. Other research directions that could be useful for analyzing the usability of polymers with potential medical applications would include morphological and volumetric analyses, such as tomographic studies.

## Figures and Tables

**Table 1:** Selected mechanical properties of polymeric materials.

Mechanical Properties	3DGence PEEK [19]	Stratasys MED610 [20]
Ultimate tensile strength (UTS) [in MPa]	105	50–65
Elongation at break [in %]	30	10–25
Young’s modulus [in MPa]	4100	2200–3200
Bending strength [in MPa]	130	75–110
Thermal properties	3DGence PEEK [19]	Stratasys MED610 [20]
Melting temperature [in °C]	343	45–50 (HDT 1.82 MPa)
Glass transition [in °C]	143	52–54
Thermal conductivity [in W m^−1^ K^−1^]	0.32	n/a

**Table 2:** Printing parameters of the various materials. * The printing speed for MED610 is automatically selected in the dedicated software for an exact geometry.

	Layer Thickness [mm]	Printing Speed	Type of Infill	Number of Contours	Infill [%]
ED610	0.03	high speed *	standard	-	100
PEEK	0.15	50 mm/s	parallel lines	5	100

**Table 3:** Surface roughness parameters of the PEEK sample.

Surface Roughness Parameters of PEEK Samples	As Built Condition	After Sterilization at 121 °C	After Sterilization at 134 °C
S_a_—arithmetic mean of the absolute values of the profile ordinates (mean roughness)	3.14 μm	2.49 μm	3.65 μm
S_q_—root mean square of the profile ordinates (RMS roughness)	4.09 μm	3.22 μm	4.86 μm
S_p_—maximum peak height (maximum summit height)	26.4 μm	18.3 μm	19.2 μm
S_v_—minimum valley depth (maximum pit depth)	14.9 μm	9.19 μm	20.5 μm
S_sk_—kkewness, which is the ratio of the mean value of the third power of the profile ordinates to the third power of the S_q_ (root mean square height).	0.453 μm	0.653 μm	−0.42 μm
S_ku_—kurtosis, which is the ratio of the mean value of the fourth power of the profile ordinates to the fourth power of the S_q_ (root mean square height).	4.10 μm	3.57 μm	4.00 μm
S_z_—maximum height of the summit to the valley, which is the sum of the maximum peak height and the maximum valley depth.	41.30 μm	27.49 μm	39.70 μm

**Table 4:** Surface roughness parameters of the MED610.

Surface Roughness Parameters of PEEK Samples	As Built Condition	After Sterilization at 121 °C	After Sterilization at 134 °C
S_a_—arithmetic mean of the absolute values of the profile ordinates (mean roughness)	3.40 μm	2.43 μm	1.08 μm
S_q_—root mean square of the profile ordinates (RMS roughness)	4.55 μm	3.18 μm	1.58 μm
S_p_—maximum peak height (maximum summit height)	26.2 μm	13.5 μm	9.73 μm
S_v_—minimum valley depth (maximum pit depth)	9.13 μm	7.93 μm	6.97 μm
S_sk_—skewness, which is the ratio of the mean value of the third power of the profile ordinates to the third power of the S_q_ (root mean square height).	1.39 μm	0.928 μm	1.11 μm
S_ku_—kurtosis, which is the ratio of the mean value of the fourth power of the profile ordinates to the fourth power of the S_q_ (root mean square height).	4.10 μm	4.03 μm	6.98 μm
S_z_—maximum height of the summit to the valley, which is the sum of the maximum peak height and the maximum valley depth.	35.33 μm	21.43 μm	16.70 μm

## Data Availability

This study did not report any data.
